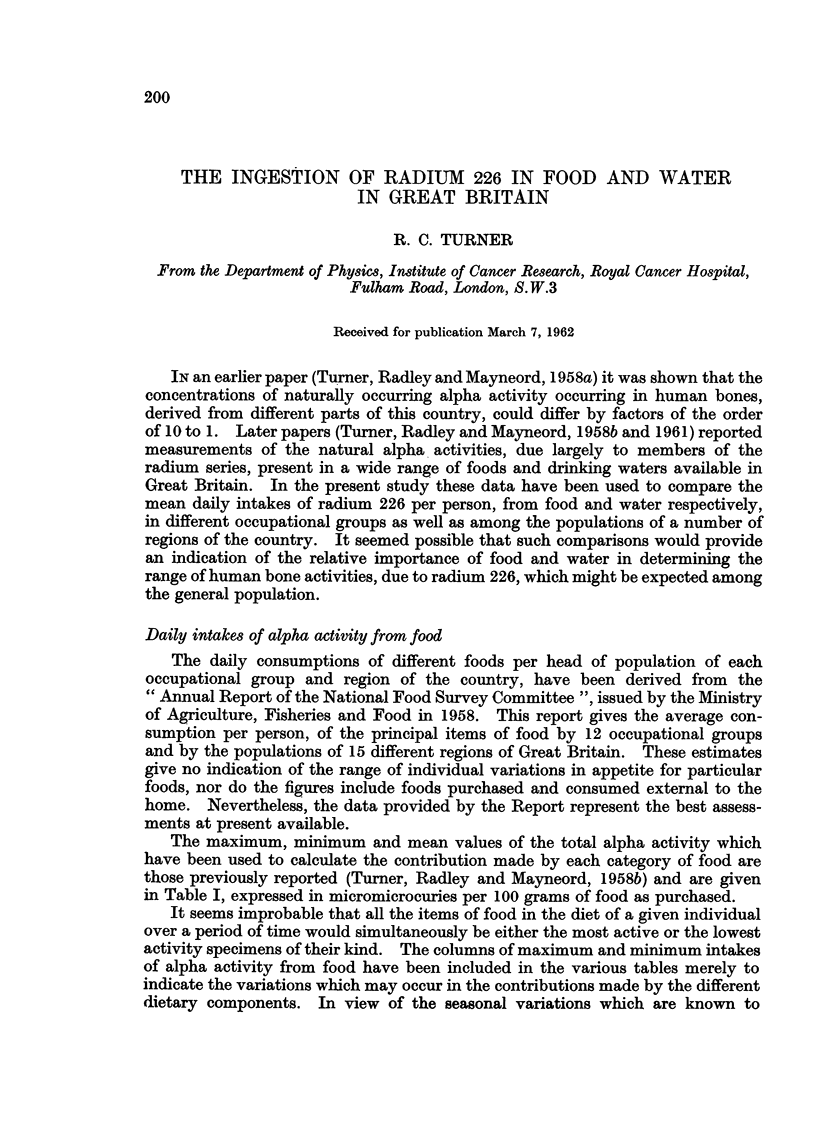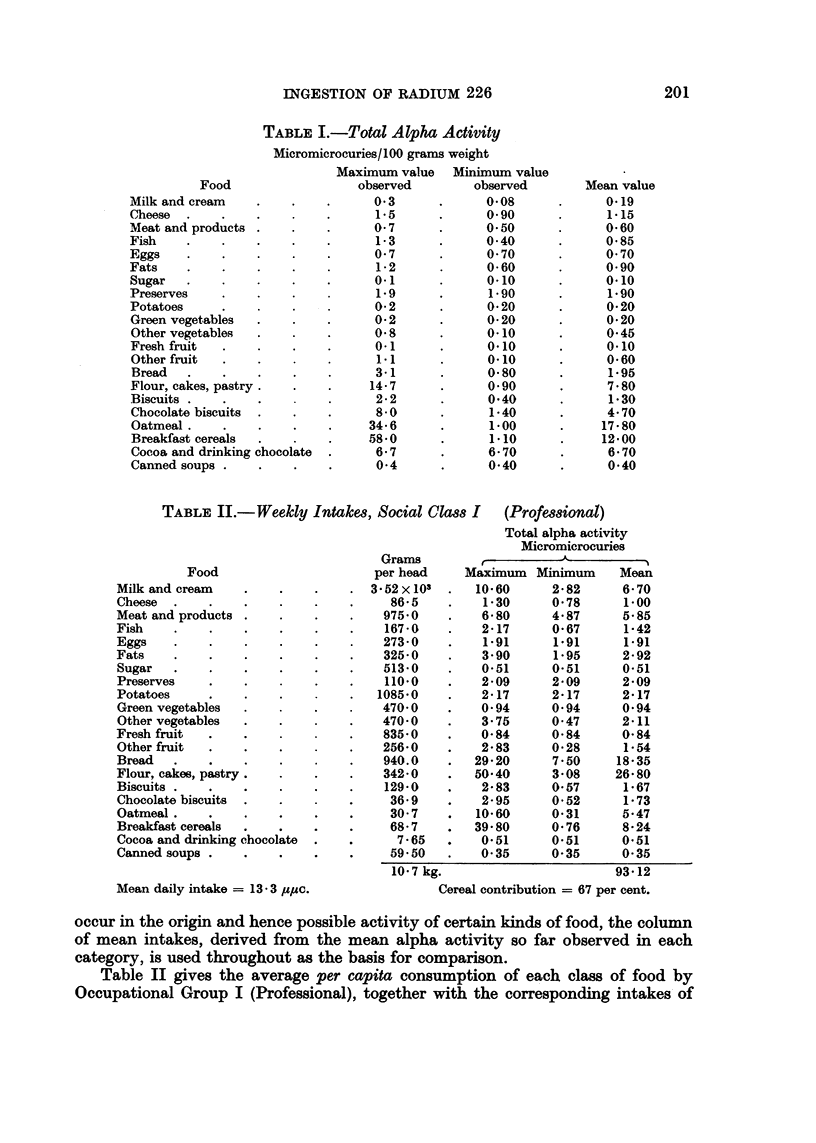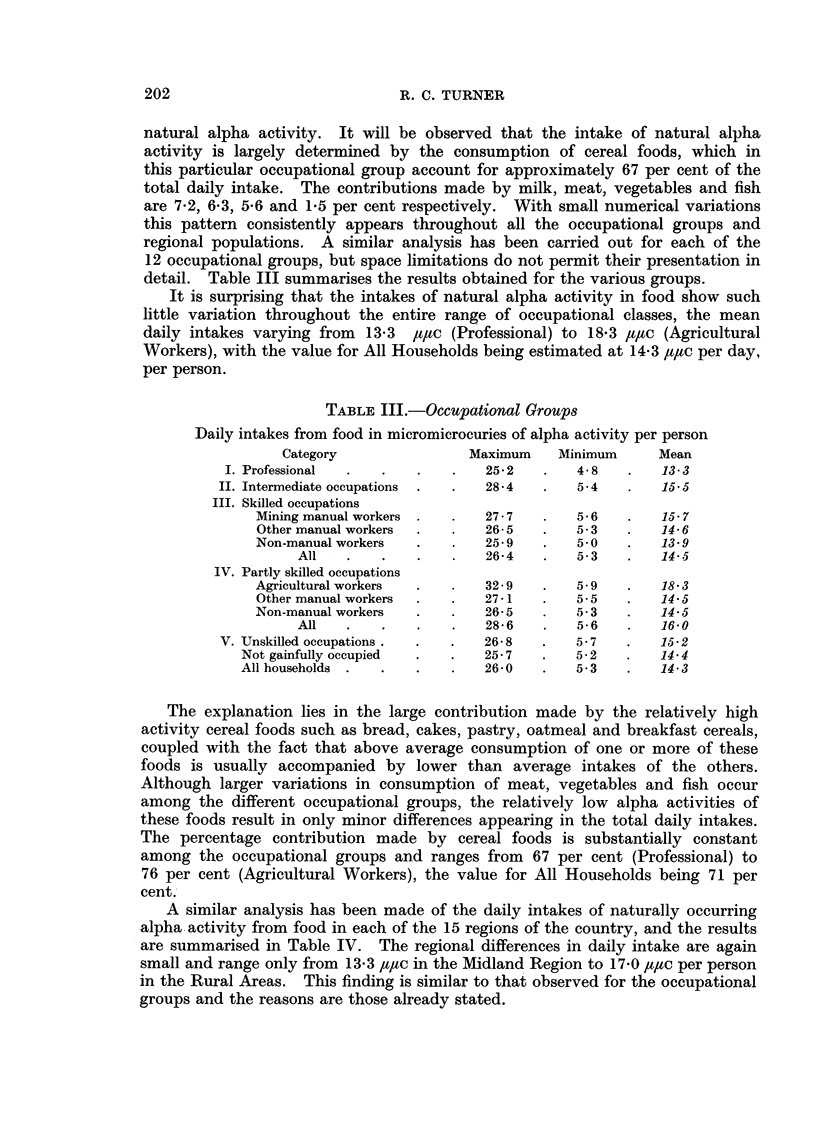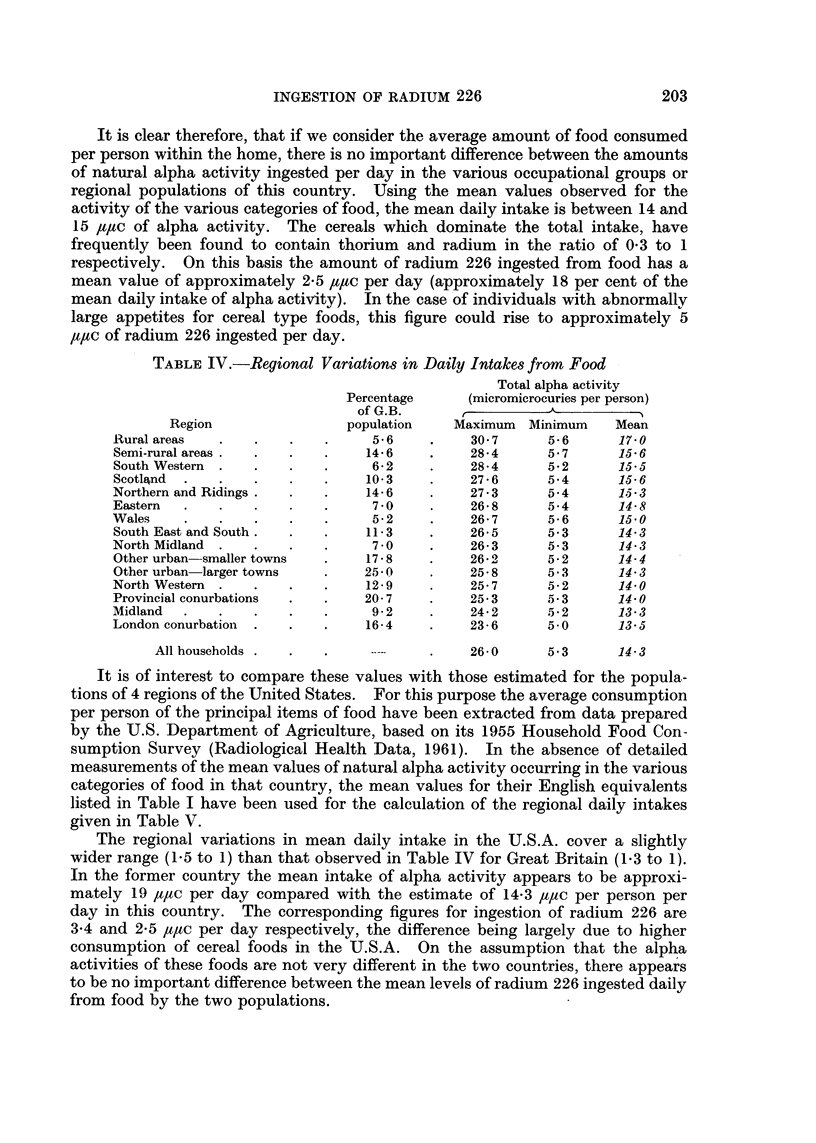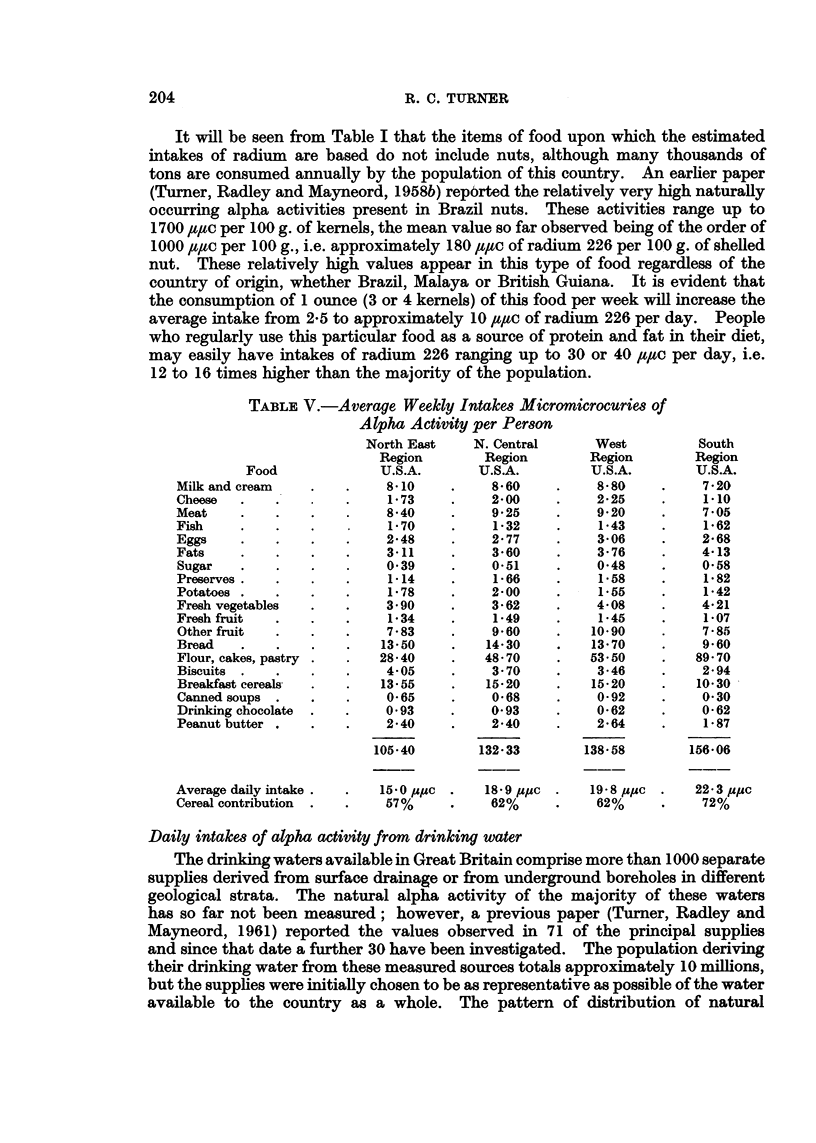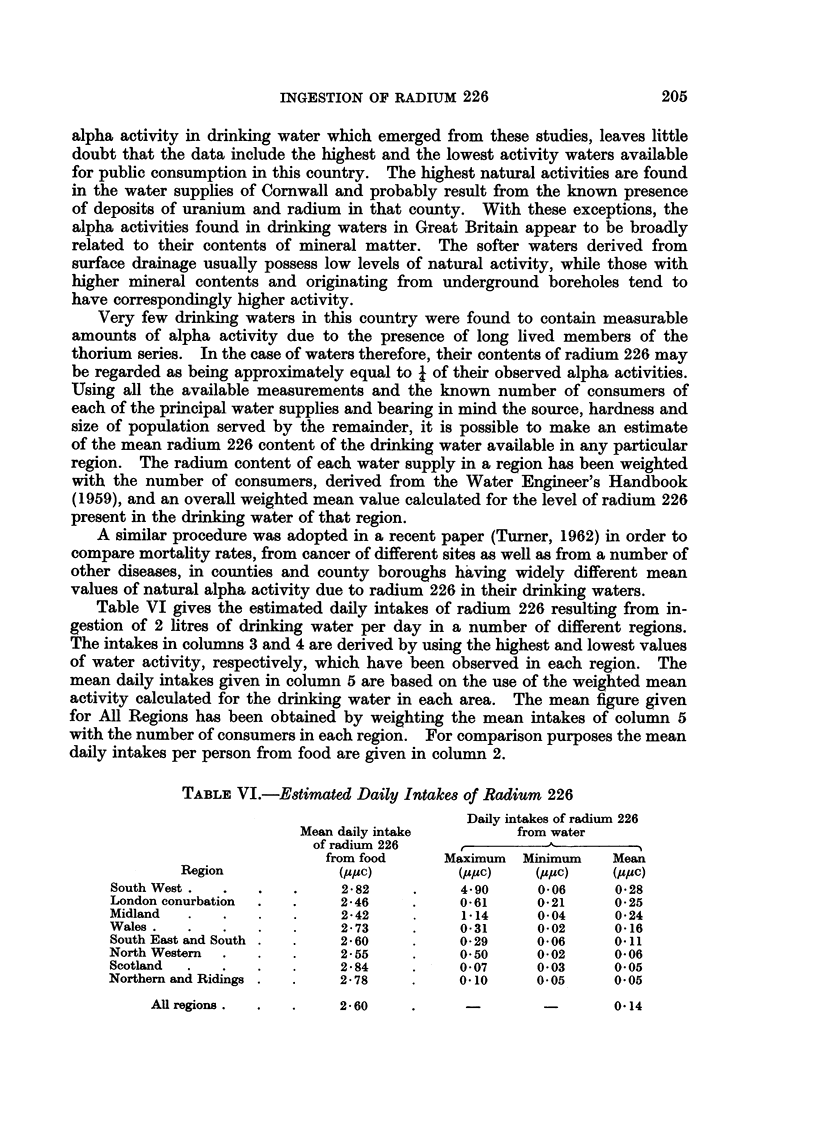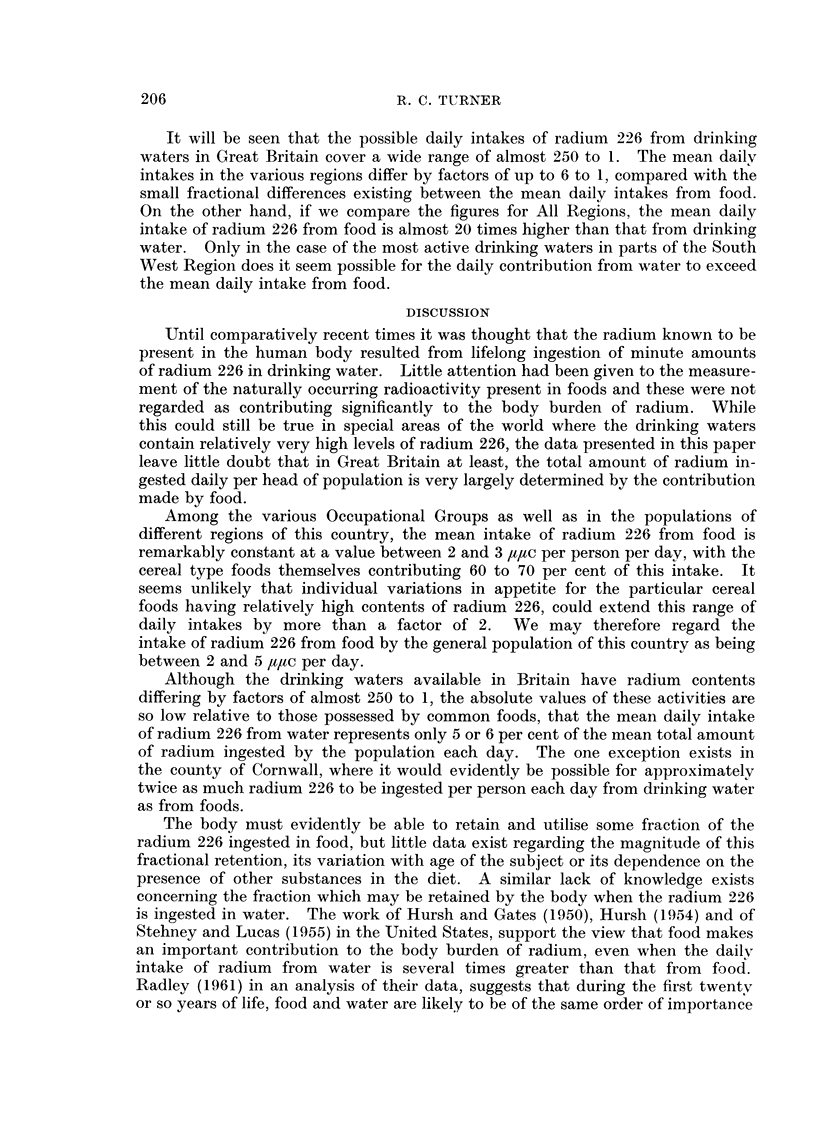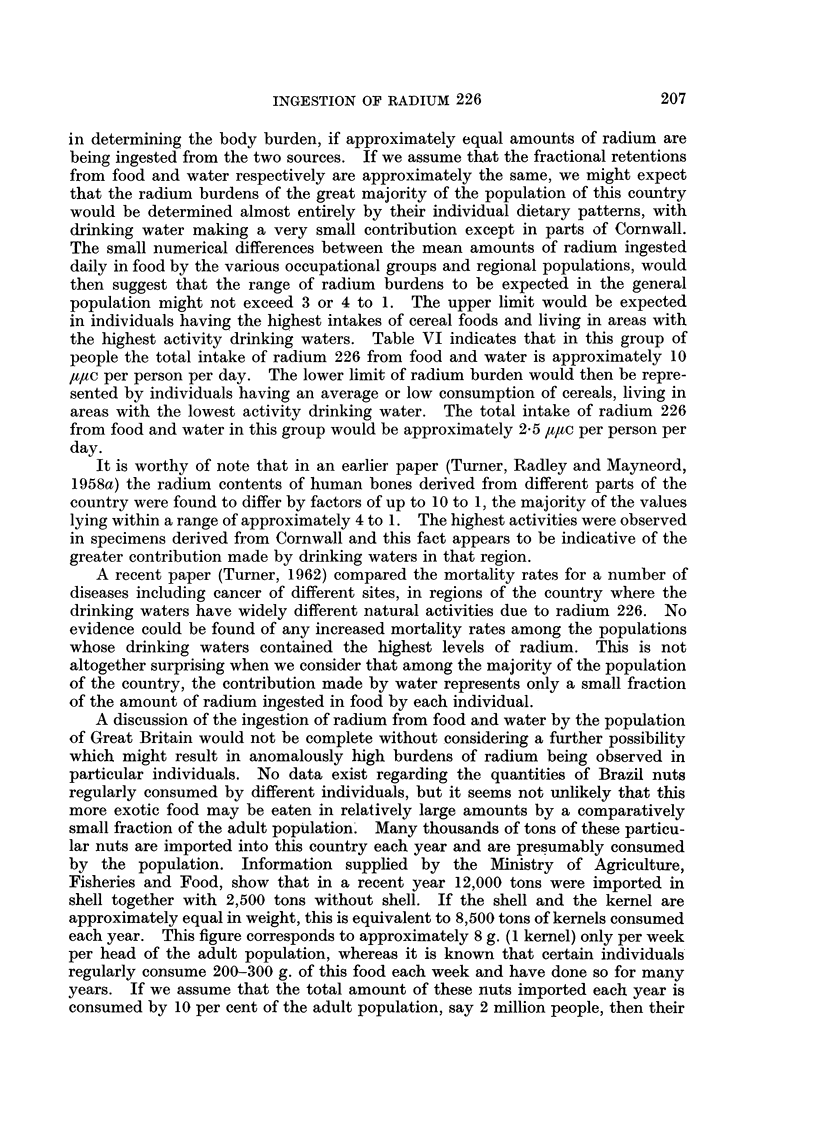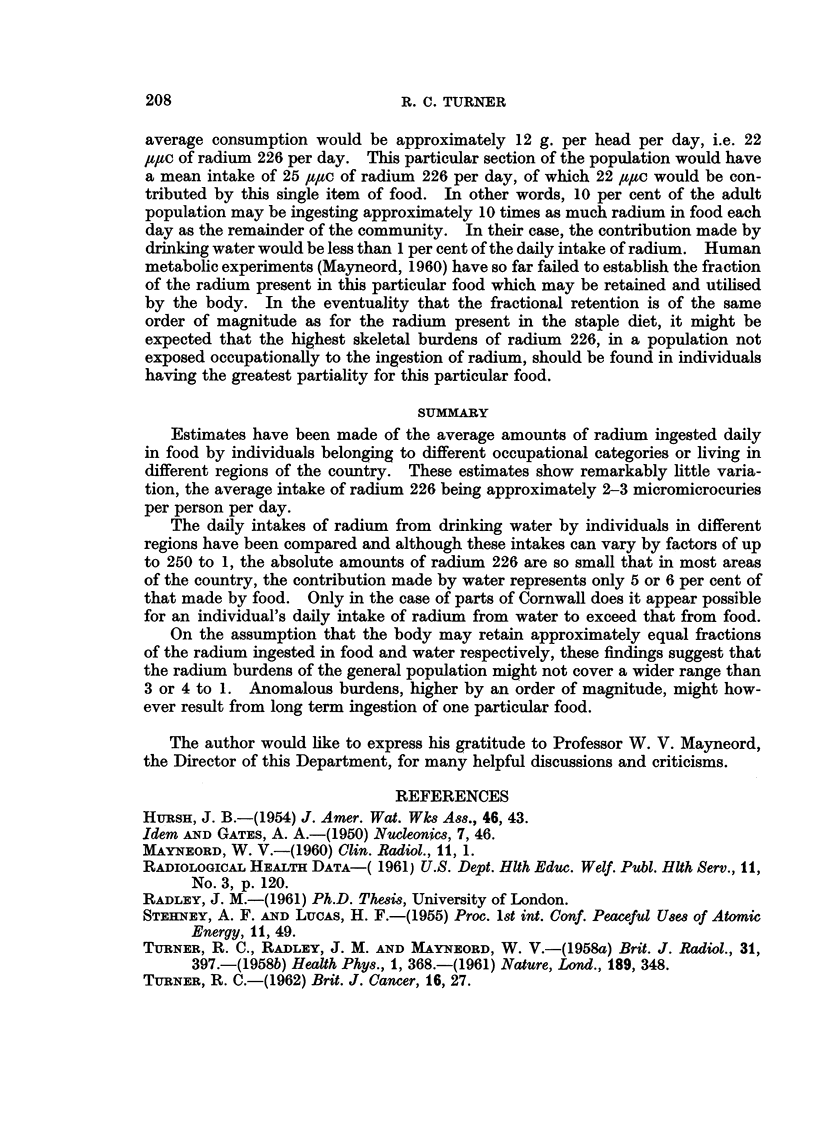# The Ingestion of Radium 226 in Food and Water in Great Britain

**DOI:** 10.1038/bjc.1962.23

**Published:** 1962-06

**Authors:** R. C. Turner


### THE INGESTION OF RADIUM 226 IN FOOD AND WATER IN GREAT BRITAIN

R. C. TURNER

*From the Department of Physics, Institute of Cancer Research, Royal Cancer Hospital, Fulham Road, London, S.W.3*

Received for publication March 7, 1962

IN an earlier paper (Turner, Radley and Mayneord, 1958*a*) it was shown that the concentrations of naturally occurring alpha activity occurring in human bones, derived from different parts of this country, could differ by factors of the order of 10 to 1. Later papers (Turner, Radley and Mayneord, 1958*b* and 1961) reported measurements of the natural alpha activities, due largely to members of the radium series, present in a wide range of foods and drinking waters available in Great Britain. In the present study these data have been used to compare the mean daily intakes of radium 226 per person, from food and water respectively, in different occupational groups as well as among the populations of a number of regions of the country. It seemed possible that such comparisons would provide an indication of the relative importance of food and water in determining the range of human bone activities, due to radium 226, which might be expected among the general population.

*Daily intakes of alpha activity from food*

The daily consumptions of different foods per head of population of each occupational group and region of the country, have been derived from the " Annual Report of the National Food Survey Committee ", issued by the Ministry of Agriculture, Fisheries and Food in 1958. This report gives the average consumption per person, of the principal items of food by 12 occupational groups and by the populations of 15 different regions of Great Britain. These estimates give no indication of the range of individual variations in appetite for particular foods, nor do the figures include foods purchased and consumed external to the home. Nevertheless, the data provided by the Report represent the best assessments at present available.

The maximum, minimum and mean values of the total alpha activity which have been used to calculate the contribution made by each category of food are those previously reported (Turner, Radley and Mayneord, 1958*b*) and are given in Table I, expressed in micromicrocuries per 100 grams of food as purchased.

It seems improbable that all the items of food in the diet of a given individual over a period of time would simultaneously be either the most active or the lowest activity specimens of their kind. The columns of maximum and minimum intakes of alpha activity from food have been included in the various tables merely to indicate the variations which may occur in the contributions made by the different dietary components. In view of the seasonal variations which are known to

TABLE I.—*Total Alpha Activity*

Micromicrocuries/100 grams weight

| Food | Maximum value observed | Minimum value observed | Mean value |
|---|---|---|---|
| Milk and cream | 0·3 | 0·08 | 0·19 |
| Cheese | 1·5 | 0·90 | 1·15 |
| Meat and products | 0·7 | 0·50 | 0·60 |
| Fish | 1·3 | 0·40 | 0·85 |
| Eggs | 0·7 | 0·70 | 0·70 |
| Fats | 1·2 | 0·60 | 0·90 |
| Sugar | 0·1 | 0·10 | 0·10 |
| Preserves | 1·9 | 1·90 | 1·90 |
| Potatoes | 0·2 | 0·20 | 0·20 |
| Green vegetables | 0·2 | 0·20 | 0·20 |
| Other vegetables | 0·8 | 0·10 | 0·45 |
| Fresh fruit | 0·1 | 0·10 | 0·10 |
| Other fruit | 1·1 | 0·10 | 0·60 |
| Bread | 3·1 | 0·80 | 1·95 |
| Flour, cakes, pastry | 14·7 | 0·90 | 7·80 |
| Biscuits | 2·2 | 0·40 | 1·30 |
| Chocolate biscuits | 8·0 | 1·40 | 4·70 |
| Oatmeal | 34·6 | 1·00 | 17·80 |
| Breakfast cereals | 58·0 | 1·10 | 12·00 |
| Cocoa and drinking chocolate | 6·7 | 6·70 | 6·70 |
| Canned soups | 0·4 | 0·40 | 0·40 |

TABLE II.—*Weekly Intakes, Social Class I (Professional)*

| Food | Grams per head | Total alpha activity Micromicrocuries: Maximum | Minimum | Mean |
|---|---|---|---|---|
| Milk and cream | $3 \cdot 52 \times 10^3$ | 10·60 | 2·82 | 6·70 |
| Cheese | 86·5 | 1·30 | 0·78 | 1·00 |
| Meat and products | 975·0 | 6·80 | 4·87 | 5·85 |
| Fish | 167·0 | 2·17 | 0·67 | 1·42 |
| Eggs | 273·0 | 1·91 | 1·91 | 1·91 |
| Fats | 325·0 | 3·90 | 1·95 | 2·92 |
| Sugar | 513·0 | 0·51 | 0·51 | 0·51 |
| Preserves | 110·0 | 2·09 | 2·09 | 2·09 |
| Potatoes | 1085·0 | 2·17 | 2·17 | 2·17 |
| Green vegetables | 470·0 | 0·94 | 0·94 | 0·94 |
| Other vegetables | 470·0 | 3·75 | 0·47 | 2·11 |
| Fresh fruit | 835·0 | 0·84 | 0·84 | 0·84 |
| Other fruit | 256·0 | 2·83 | 0·28 | 1·54 |
| Bread | 940.0 | 29·20 | 7·50 | 18·35 |
| Flour, cakes, pastry | 342·0 | 50·40 | 3·08 | 26·80 |
| Biscuits | 129·0 | 2·83 | 0·57 | 1·67 |
| Chocolate biscuits | 36·9 | 2·95 | 0·52 | 1·73 |
| Oatmeal | 30·7 | 10·60 | 0·31 | 5·47 |
| Breakfast cereals | 68·7 | 39·80 | 0·76 | 8·24 |
| Cocoa and drinking chocolate | 7·65 | 0·51 | 0·51 | 0·51 |
| Canned soups | 59·50 | 0·35 | 0·35 | 0·35 |
| | 10·7 kg. | | | 93·12 |

Mean daily intake = 13·3 $\mu\mu$c. Cereal contribution = 67 per cent.

occur in the origin and hence possible activity of certain kinds of food, the column of mean intakes, derived from the mean alpha activity so far observed in each category, is used throughout as the basis for comparison.

Table II gives the average *per capita* consumption of each class of food by Occupational Group I (Professional), together with the corresponding intakes of natural alpha activity. It will be observed that the intake of natural alpha activity is largely determined by the consumption of cereal foods, which in this particular occupational group account for approximately 67 per cent of the total daily intake. The contributions made by milk, meat, vegetables and fish are 7·2, 6·3, 5·6 and 1·5 per cent respectively. With small numerical variations this pattern consistently appears throughout all the occupational groups and regional populations. A similar analysis has been carried out for each of the 12 occupational groups, but space limitations do not permit their presentation in detail. Table III summarises the results obtained for the various groups.

It is surprising that the intakes of natural alpha activity in food show such little variation throughout the entire range of occupational classes, the mean daily intakes varying from 13·3 $\mu\mu c$ (Professional) to 18·3 $\mu\mu c$ (Agricultural Workers), with the value for All Households being estimated at 14·3 $\mu\mu c$ per day, per person.

TABLE III.—*Occupational Groups*

Daily intakes from food in micromicrocuries of alpha activity per person

| Category | Maximum | Minimum | Mean |
|---|---|---|---|
| I. Professional | 25·2 | 4·8 | *13·3* |
| II. Intermediate occupations | 28·4 | 5·4 | *15·5* |
| III. Skilled occupations | | | |
| Mining manual workers | 27·7 | 5·6 | *15·7* |
| Other manual workers | 26·5 | 5·3 | *14·6* |
| Non-manual workers | 25·9 | 5·0 | *13·9* |
| All | 26·4 | 5·3 | *14·5* |
| IV. Partly skilled occupations | | | |
| Agricultural workers | 32·9 | 5·9 | *18·3* |
| Other manual workers | 27·1 | 5·5 | *14·5* |
| Non-manual workers | 26·5 | 5·3 | *14·5* |
| All | 28·6 | 5·6 | *16·0* |
| V. Unskilled occupations | 26·8 | 5·7 | *15·2* |
| Not gainfully occupied | 25·7 | 5·2 | *14·4* |
| All households | 26·0 | 5·3 | *14·3* |

The explanation lies in the large contribution made by the relatively high activity cereal foods such as bread, cakes, pastry, oatmeal and breakfast cereals, coupled with the fact that above average consumption of one or more of these foods is usually accompanied by lower than average intakes of the others. Although larger variations in consumption of meat, vegetables and fish occur among the different occupational groups, the relatively low alpha activities of these foods result in only minor differences appearing in the total daily intakes. The percentage contribution made by cereal foods is substantially constant among the occupational groups and ranges from 67 per cent (Professional) to 76 per cent (Agricultural Workers), the value for All Households being 71 per cent.

A similar analysis has been made of the daily intakes of naturally occurring alpha activity from food in each of the 15 regions of the country, and the results are summarised in Table IV. The regional differences in daily intake are again small and range only from 13·3 $\mu\mu c$ in the Midland Region to 17·0 $\mu\mu c$ per person in the Rural Areas. This finding is similar to that observed for the occupational groups and the reasons are those already stated.

It is clear therefore, that if we consider the average amount of food consumed per person within the home, there is no important difference between the amounts of natural alpha activity ingested per day in the various occupational groups or regional populations of this country. Using the mean values observed for the activity of the various categories of food, the mean daily intake is between 14 and 15 $\mu\mu c$ of alpha activity. The cereals which dominate the total intake, have frequently been found to contain thorium and radium in the ratio of 0·3 to 1 respectively. On this basis the amount of radium 226 ingested from food has a mean value of approximately 2·5 $\mu\mu c$ per day (approximately 18 per cent of the mean daily intake of alpha activity). In the case of individuals with abnormally large appetites for cereal type foods, this figure could rise to approximately 5 $\mu\mu c$ of radium 226 ingested per day.

TABLE IV.—*Regional Variations in Daily Intakes from Food*

| Region | Percentage of G.B. population | Total alpha activity (micromicrocuries per person) Maximum | Minimum | Mean |
|---|---|---|---|---|
| Rural areas | 5·6 | 30·7 | 5·6 | *17·0* |
| Semi-rural areas | 14·6 | 28·4 | 5·7 | *15·6* |
| South Western | 6·2 | 28·4 | 5·2 | *15·5* |
| Scotland | 10·3 | 27·6 | 5·4 | *15·6* |
| Northern and Ridings | 14·6 | 27·3 | 5·4 | *15·3* |
| Eastern | 7·0 | 26·8 | 5·4 | *14·8* |
| Wales | 5·2 | 26·7 | 5·6 | *15·0* |
| South East and South | 11·3 | 26·5 | 5·3 | *14·3* |
| North Midland | 7·0 | 26·3 | 5·3 | *14·3* |
| Other urban—smaller towns | 17·8 | 26·2 | 5·2 | *14·4* |
| Other urban—larger towns | 25·0 | 25·8 | 5·3 | *14·3* |
| North Western | 12·9 | 25·7 | 5·2 | *14·0* |
| Provincial conurbations | 20·7 | 25·3 | 5·3 | *14·0* |
| Midland | 9·2 | 24·2 | 5·2 | *13·3* |
| London conurbation | 16·4 | 23·6 | 5·0 | *13·5* |
| All households | — | 26·0 | 5·3 | *14·3* |

It is of interest to compare these values with those estimated for the populations of 4 regions of the United States. For this purpose the average consumption per person of the principal items of food have been extracted from data prepared by the U.S. Department of Agriculture, based on its 1955 Household Food Consumption Survey (Radiological Health Data, 1961). In the absence of detailed measurements of the mean values of natural alpha activity occurring in the various categories of food in that country, the mean values for their English equivalents listed in Table I have been used for the calculation of the regional daily intakes given in Table V.

The regional variations in mean daily intake in the U.S.A. cover a slightly wider range (1·5 to 1) than that observed in Table IV for Great Britain (1·3 to 1). In the former country the mean intake of alpha activity appears to be approximately 19 $\mu\mu c$ per day compared with the estimate of 14·3 $\mu\mu c$ per person per day in this country. The corresponding figures for ingestion of radium 226 are 3·4 and 2·5 $\mu\mu c$ per day respectively, the difference being largely due to higher consumption of cereal foods in the U.S.A. On the assumption that the alpha activities of these foods are not very different in the two countries, there appears to be no important difference between the mean levels of radium 226 ingested daily from food by the two populations.

It will be seen from Table I that the items of food upon which the estimated intakes of radium are based do not include nuts, although many thousands of tons are consumed annually by the population of this country. An earlier paper (Turner, Radley and Mayneord, 1958*b*) reported the relatively very high naturally occurring alpha activities present in Brazil nuts. These activities range up to 1700 $\mu\mu$c per 100 g. of kernels, the mean value so far observed being of the order of 1000 $\mu\mu$c per 100 g., i.e. approximately 180 $\mu\mu$c of radium 226 per 100 g. of shelled nut. These relatively high values appear in this type of food regardless of the country of origin, whether Brazil, Malaya or British Guiana. It is evident that the consumption of 1 ounce (3 or 4 kernels) of this food per week will increase the average intake from 2·5 to approximately 10 $\mu\mu$c of radium 226 per day. People who regularly use this particular food as a source of protein and fat in their diet, may easily have intakes of radium 226 ranging up to 30 or 40 $\mu\mu$c per day, i.e. 12 to 16 times higher than the majority of the population.

TABLE V.—*Average Weekly Intakes Micromicrocuries of Alpha Activity per Person*

| Food | North East Region U.S.A. | N. Central Region U.S.A. | West Region U.S.A. | South Region U.S.A. |
|---|---|---|---|---|
| Milk and cream | 8·10 | 8·60 | 8·80 | 7·20 |
| Cheese | 1·73 | 2·00 | 2·25 | 1·10 |
| Meat | 8·40 | 9·25 | 9·20 | 7·05 |
| Fish | 1·70 | 1·32 | 1·43 | 1·62 |
| Eggs | 2·48 | 2·77 | 3·06 | 2·68 |
| Fats | 3·11 | 3·60 | 3·76 | 4·13 |
| Sugar | 0·39 | 0·51 | 0·48 | 0·58 |
| Preserves | 1·14 | 1·66 | 1·58 | 1·82 |
| Potatoes | 1·78 | 2·00 | 1·55 | 1·42 |
| Fresh vegetables | 3·90 | 3·62 | 4·08 | 4·21 |
| Fresh fruit | 1·34 | 1·49 | 1·45 | 1·07 |
| Other fruit | 7·83 | 9·60 | 10·90 | 7·85 |
| Bread | 13·50 | 14·30 | 13·70 | 9·60 |
| Flour, cakes, pastry | 28·40 | 48·70 | 53·50 | 89·70 |
| Biscuits | 4·05 | 3·70 | 3·46 | 2·94 |
| Breakfast cereals | 13·55 | 15·20 | 15·20 | 10·30 |
| Canned soups | 0·65 | 0·68 | 0·92 | 0·30 |
| Drinking chocolate | 0·93 | 0·93 | 0·62 | 0·62 |
| Peanut butter | 2·40 | 2·40 | 2·64 | 1·87 |
| | 105·40 | 132·33 | 138·58 | 156·06 |
| Average daily intake | 15·0 $\mu\mu$c | 18·9 $\mu\mu$c | 19·8 $\mu\mu$c | 22·3 $\mu\mu$c |
| Cereal contribution | 57% | 62% | 62% | 72% |

*Daily intakes of alpha activity from drinking water*

The drinking waters available in Great Britain comprise more than 1000 separate supplies derived from surface drainage or from underground boreholes in different geological strata. The natural alpha activity of the majority of these waters has so far not been measured; however, a previous paper (Turner, Radley and Mayneord, 1961) reported the values observed in 71 of the principal supplies and since that date a further 30 have been investigated. The population deriving their drinking water from these measured sources totals approximately 10 millions, but the supplies were initially chosen to be as representative as possible of the water available to the country as a whole. The pattern of distribution of natural alpha activity in drinking water which emerged from these studies, leaves little doubt that the data include the highest and the lowest activity waters available for public consumption in this country. The highest natural activities are found in the water supplies of Cornwall and probably result from the known presence of deposits of uranium and radium in that county. With these exceptions, the alpha activities found in drinking waters in Great Britain appear to be broadly related to their contents of mineral matter. The softer waters derived from surface drainage usually possess low levels of natural activity, while those with higher mineral contents and originating from underground boreholes tend to have correspondingly higher activity.

Very few drinking waters in this country were found to contain measurable amounts of alpha activity due to the presence of long lived members of the thorium series. In the case of waters therefore, their contents of radium 226 may be regarded as being approximately equal to $\frac{1}{4}$ of their observed alpha activities. Using all the available measurements and the known number of consumers of each of the principal water supplies and bearing in mind the source, hardness and size of population served by the remainder, it is possible to make an estimate of the mean radium 226 content of the drinking water available in any particular region. The radium content of each water supply in a region has been weighted with the number of consumers, derived from the Water Engineer's Handbook (1959), and an overall weighted mean value calculated for the level of radium 226 present in the drinking water of that region.

A similar procedure was adopted in a recent paper (Turner, 1962) in order to compare mortality rates, from cancer of different sites as well as from a number of other diseases, in counties and county boroughs having widely different mean values of natural alpha activity due to radium 226 in their drinking waters.

Table VI gives the estimated daily intakes of radium 226 resulting from ingestion of 2 litres of drinking water per day in a number of different regions. The intakes in columns 3 and 4 are derived by using the highest and lowest values of water activity, respectively, which have been observed in each region. The mean daily intakes given in column 5 are based on the use of the weighted mean activity calculated for the drinking water in each area. The mean figure given for All Regions has been obtained by weighting the mean intakes of column 5 with the number of consumers in each region. For comparison purposes the mean daily intakes per person from food are given in column 2.

TABLE VI.—*Estimated Daily Intakes of Radium* 226

| Region | Mean daily intake of radium 226 from food ($\mu\mu c$) | Daily intakes of radium 226 from water | | |
|---|---|---|---|---|
| | | Maximum ($\mu\mu c$) | Minimum ($\mu\mu c$) | Mean ($\mu\mu c$) |
| South West | 2·82 | 4·90 | 0·06 | 0·28 |
| London conurbation | 2·46 | 0·61 | 0·21 | 0·25 |
| Midland | 2·42 | 1·14 | 0·04 | 0·24 |
| Wales | 2·73 | 0·31 | 0·02 | 0·16 |
| South East and South | 2·60 | 0·29 | 0·06 | 0·11 |
| North Western | 2·55 | 0·50 | 0·02 | 0·06 |
| Scotland | 2·84 | 0·07 | 0·03 | 0·05 |
| Northern and Ridings | 2·78 | 0·10 | 0·05 | 0·05 |
| All regions | 2·60 | — | — | 0·14 |

It will be seen that the possible daily intakes of radium 226 from drinking waters in Great Britain cover a wide range of almost 250 to 1. The mean daily intakes in the various regions differ by factors of up to 6 to 1, compared with the small fractional differences existing between the mean daily intakes from food. On the other hand, if we compare the figures for All Regions, the mean daily intake of radium 226 from food is almost 20 times higher than that from drinking water. Only in the case of the most active drinking waters in parts of the South West Region does it seem possible for the daily contribution from water to exceed the mean daily intake from food.

### DISCUSSION

Until comparatively recent times it was thought that the radium known to be present in the human body resulted from lifelong ingestion of minute amounts of radium 226 in drinking water. Little attention had been given to the measurement of the naturally occurring radioactivity present in foods and these were not regarded as contributing significantly to the body burden of radium. While this could still be true in special areas of the world where the drinking waters contain relatively very high levels of radium 226, the data presented in this paper leave little doubt that in Great Britain at least, the total amount of radium ingested daily per head of population is very largely determined by the contribution made by food.

Among the various Occupational Groups as well as in the populations of different regions of this country, the mean intake of radium 226 from food is remarkably constant at a value between 2 and 3 $\mu\mu$c per person per day, with the cereal type foods themselves contributing 60 to 70 per cent of this intake. It seems unlikely that individual variations in appetite for the particular cereal foods having relatively high contents of radium 226, could extend this range of daily intakes by more than a factor of 2. We may therefore regard the intake of radium 226 from food by the general population of this country as being between 2 and 5 $\mu\mu$c per day.

Although the drinking waters available in Britain have radium contents differing by factors of almost 250 to 1, the absolute values of these activities are so low relative to those possessed by common foods, that the mean daily intake of radium 226 from water represents only 5 or 6 per cent of the mean total amount of radium ingested by the population each day. The one exception exists in the county of Cornwall, where it would evidently be possible for approximately twice as much radium 226 to be ingested per person each day from drinking water as from foods.

The body must evidently be able to retain and utilise some fraction of the radium 226 ingested in food, but little data exist regarding the magnitude of this fractional retention, its variation with age of the subject or its dependence on the presence of other substances in the diet. A similar lack of knowledge exists concerning the fraction which may be retained by the body when the radium 226 is ingested in water. The work of Hursh and Gates (1950), Hursh (1954) and of Stehney and Lucas (1955) in the United States, support the view that food makes an important contribution to the body burden of radium, even when the daily intake of radium from water is several times greater than that from food. Radley (1961) in an analysis of their data, suggests that during the first twenty or so years of life, food and water are likely to be of the same order of importance in determining the body burden, if approximately equal amounts of radium are being ingested from the two sources. If we assume that the fractional retentions from food and water respectively are approximately the same, we might expect that the radium burdens of the great majority of the population of this country would be determined almost entirely by their individual dietary patterns, with drinking water making a very small contribution except in parts of Cornwall. The small numerical differences between the mean amounts of radium ingested daily in food by the various occupational groups and regional populations, would then suggest that the range of radium burdens to be expected in the general population might not exceed 3 or 4 to 1. The upper limit would be expected in individuals having the highest intakes of cereal foods and living in areas with the highest activity drinking waters. Table VI indicates that in this group of people the total intake of radium 226 from food and water is approximately 10 $\mu\mu$c per person per day. The lower limit of radium burden would then be represented by individuals having an average or low consumption of cereals, living in areas with the lowest activity drinking water. The total intake of radium 226 from food and water in this group would be approximately 2·5 $\mu\mu$c per person per day.

It is worthy of note that in an earlier paper (Turner, Radley and Mayneord, 1958*a*) the radium contents of human bones derived from different parts of the country were found to differ by factors of up to 10 to 1, the majority of the values lying within a range of approximately 4 to 1. The highest activities were observed in specimens derived from Cornwall and this fact appears to be indicative of the greater contribution made by drinking waters in that region.

A recent paper (Turner, 1962) compared the mortality rates for a number of diseases including cancer of different sites, in regions of the country where the drinking waters have widely different natural activities due to radium 226. No evidence could be found of any increased mortality rates among the populations whose drinking waters contained the highest levels of radium. This is not altogether surprising when we consider that among the majority of the population of the country, the contribution made by water represents only a small fraction of the amount of radium ingested in food by each individual.

A discussion of the ingestion of radium from food and water by the population of Great Britain would not be complete without considering a further possibility which might result in anomalously high burdens of radium being observed in particular individuals. No data exist regarding the quantities of Brazil nuts regularly consumed by different individuals, but it seems not unlikely that this more exotic food may be eaten in relatively large amounts by a comparatively small fraction of the adult population. Many thousands of tons of these particular nuts are imported into this country each year and are presumably consumed by the population. Information supplied by the Ministry of Agriculture, Fisheries and Food, show that in a recent year 12,000 tons were imported in shell together with 2,500 tons without shell. If the shell and the kernel are approximately equal in weight, this is equivalent to 8,500 tons of kernels consumed each year. This figure corresponds to approximately 8 g. (1 kernel) only per week per head of the adult population, whereas it is known that certain individuals regularly consume 200–300 g. of this food each week and have done so for many years. If we assume that the total amount of these nuts imported each year is consumed by 10 per cent of the adult population, say 2 million people, then their average consumption would be approximately 12 g. per head per day, i.e. 22 $\mu\mu c$ of radium 226 per day. This particular section of the population would have a mean intake of 25 $\mu\mu c$ of radium 226 per day, of which 22 $\mu\mu c$ would be contributed by this single item of food. In other words, 10 per cent of the adult population may be ingesting approximately 10 times as much radium in food each day as the remainder of the community. In their case, the contribution made by drinking water would be less than 1 per cent of the daily intake of radium. Human metabolic experiments (Mayneord, 1960) have so far failed to establish the fraction of the radium present in this particular food which may be retained and utilised by the body. In the eventuality that the fractional retention is of the same order of magnitude as for the radium present in the staple diet, it might be expected that the highest skeletal burdens of radium 226, in a population not exposed occupationally to the ingestion of radium, should be found in individuals having the greatest partiality for this particular food.

### SUMMARY

Estimates have been made of the average amounts of radium ingested daily in food by individuals belonging to different occupational categories or living in different regions of the country. These estimates show remarkably little variation, the average intake of radium 226 being approximately 2–3 micromicrocuries per person per day.

The daily intakes of radium from drinking water by individuals in different regions have been compared and although these intakes can vary by factors of up to 250 to 1, the absolute amounts of radium 226 are so small that in most areas of the country, the contribution made by water represents only 5 or 6 per cent of that made by food. Only in the case of parts of Cornwall does it appear possible for an individual's daily intake of radium from water to exceed that from food.

On the assumption that the body may retain approximately equal fractions of the radium ingested in food and water respectively, these findings suggest that the radium burdens of the general population might not cover a wider range than 3 or 4 to 1. Anomalous burdens, higher by an order of magnitude, might however result from long term ingestion of one particular food.

The author would like to express his gratitude to Professor W. V. Mayneord, the Director of this Department, for many helpful discussions and criticisms.

### REFERENCES

HURSH, J. B.—(1954) *J. Amer. Wat. Wks Ass.*, **46**, 43.
*Idem* AND GATES, A. A.—(1950) *Nucleonics*, 7, 46.
MAYNEORD, W. V.—(1960) *Clin. Radiol.*, **11**, 1.
RADIOLOGICAL HEALTH DATA—( 1961) *U.S. Dept. Hlth Educ. Welf. Publ. Hlth Serv.*, **11**, No. 3, p. 120.
RADLEY, J. M.—(1961) *Ph.D. Thesis*, University of London.
STEHNEY, A. F. AND LUCAS, H. F.—(1955) *Proc. 1st int. Conf. Peaceful Uses of Atomic Energy*, **11**, 49.
TURNER, R. C., RADLEY, J. M. AND MAYNEORD, W. V.—(1958*a*) *Brit. J. Radiol.*, **31**, 397.—(1958*b*) *Health Phys.*, **1**, 368.—(1961) *Nature, Lond.*, **189**, 348.
TURNER, R. C.—(1962) *Brit. J. Cancer*, **16**, 27.